# Mechanistic roles and clinical applications of tertiary lymphoid structures in colorectal cancer drug resistance

**DOI:** 10.20517/cdr.2026.35

**Published:** 2026-07-28

**Authors:** Zhiliang Li, Wenqing Jia, Zichao Guo, Fang Zheng, Chenhao Huang, Tao Zhang, Zhuoqing Xu, Haoran Feng, Xi Cheng, Ren Zhao

**Affiliations:** ^1^Department of General Surgery, Ruijin Hospital, Shanghai Jiao Tong University School of Medicine, Shanghai 200025, China.; ^2^Shanghai Institute of Digestive Surgery, Ruijin Hospital, Shanghai Jiao Tong University School of Medicine, Shanghai 200025, China.; ^#^These authors contributed equally.

**Keywords:** Tertiary lymphoid structures, colorectal cancer, tumor microenvironment, tumor-infiltrating lymphocytes, drug resistance, immunotherapy

## Abstract

Therapeutic resistance represents a formidable bottleneck in colorectal cancer (CRC) management, severely limiting the clinical efficacy of both conventional regimens and novel immunotherapies. Emerging evidence underscores tertiary lymphoid structure (TLS) - ectopic lymphoid aggregates newly sculpted within the tumor microenvironment (TME) - as pivotal orchestrators of localized antitumor immunity and prime therapeutic targets to circumvent resistance. Distinct from secondary lymphoid organs, TLS facilitate *in situ* immune cell priming, clonal expansion, and functional differentiation, thereby sustaining a robust adaptive immune response. This review systematically dissects the cellular architecture, maturation dynamics, and spatial heterogeneity of TLS in primary and metastatic CRC, clarifying how these attributes dictate clinical outcomes and treatment responsiveness. Mechanistically, we delineate the molecular networks driving TLS neogenesis, encompassing chemokine cascades, tumor necrosis factor (TNF) superfamily signaling, and interactive crosstalk with the gut microbiota. Furthermore, we summarize cutting-edge preclinical strategies - including stimulator of interferon genes agonists, bio-nanovaccines, and hypofractionated radiotherapy combinations - engineered to trigger TLS neogenesis, promote germinal center (GC) maturation, and enrich stem-like effector tumor-infiltrating lymphocytes (TILs) to convert immunologically “cold” tumors into “hot” niches. By integrating mechanistic paradigms with clinical applications, this review provides a definitive framework for leveraging TLS targeting as a transformative modality to modulate drug resistance and optimize therapeutic trajectories in CRC; the graphical abstract is shown below.

## INTRODUCTION

Colorectal cancer (CRC) remains the third most common malignancy and the second-leading cause of cancer-related mortality worldwide, with over 1.9 million new CRC cases and 930,000 deaths annually^[1]^. Despite decades of clinical exploration and therapeutic advancements, including surgery, chemotherapy, and radiotherapy, the overall survival (OS) of CRC patients still necessitates further improvement through personalized and targeted anticancer strategies^[2,3]^. In the emerging era of immunotherapy, immune checkpoint inhibitors (ICIs) targeting CTLA-4 or the PD-1/PD-L1 pathway have demonstrated promising efficacy and favorable tolerability^[4-6]^. However, the clinical benefit of ICIs is predominantly observed in the 15%-20% of CRC patients with microsatellite instability-high (MSI-H) status, a consequence of DNA mismatch repair deficiency (dMMR), which is associated with an immunologically active tumor microenvironment (TME) and high tumor mutational burden (TMB) that collectively foster robust anti-tumor immunity compared with proficient mismatch repair-proficient (pMMR) tumors^[7,8]^. Consequently, there is an urgent need to identify predictive biomarkers and develop effective strategies to overcome therapeutic resistance, particularly among the majority of patients with microsatellite-stable (MSS) tumors who derive limited benefit from current immunotherapies.

Consequently, tertiary lymphoid structures (TLSs) have attracted increasing attention as important regulators of the TME. TLS are ectopic lymphoid aggregates that arise in non-lymphoid tissues under conditions of chronic inflammation, including persistent infections, autoimmune diseases, transplant rejection, and malignancies^[9]^. Structurally analogous to secondary lymphoid organs (SLOs), TLS is composed of a diverse array of immune cells, including T cells, B cells, dendritic cells (DCs), follicular dendritic cells (FDCs), myeloid cells, high endothelial venules (HEVs), and fibroblasts, all of which contribute to their formation and function^[10,11]^. Functionally, TLS serves as a privileged site for local tumor antigen presentation by DCs and B cells, enabling the rapid initiation of innate and adaptive anti-tumor immune responses while providing a supportive niche for T- and B-cell proliferation and differentiation. The presence of TLS has been associated with reduced recurrence rates, improved prognosis, and enhanced responsiveness to immunotherapy across multiple cancer types, including melanoma, soft tissue sarcoma, renal cell carcinoma, urothelial carcinoma, and CRC^[12-16]^. Although the classification of TLS maturation remains a subject of ongoing discussion, mature TLS - characterized by the presence of germinal centers (GCs) and organized B-cell follicles with associated T-cell zones - is generally considered to confer superior prognostic value compared with immature or single-zone TLS^[17,18]^. Furthermore, TLS exhibits considerable heterogeneity in spatial distribution, occurring within intratumoral regions, at the invasive margin, or in peritumoral areas, with varying densities across different CRC tumor sites. This heterogeneity in maturation status, localization, and density contributes to diverse immunological functions and distinct clinical outcomes in CRC patients^[19-21]^.

Given the established association between TLS and improved immune responses, emerging evidence suggests that TLS may play a pivotal role in modulating drug resistance in CRC. In this review, we aim to provide a comprehensive framework for understanding the functional and clinical relevance of TLS, with a particular focus on their relationship with therapeutic resistance. We systematically summarize the composition, formation, and maturation of TLS in CRC, and elaborate on their clinical implications in modulating responses to chemotherapy, immunotherapy, and other cutting-edge treatment modalities, thereby offering insights into how TLS-based strategies may help overcome drug resistance and improve patient outcomes.

Literature for this review was retrieved from databases including PubMed and Web of Science (2010 - March 2026) using relevant keywords, and the inclusion/exclusion criteria (original research, reviews, and clinical trials in English; studies focusing on TLS in solid tumors, with priority given to CRC). The aim of this review is to synthesize current knowledge and provide a forward-looking perspective, rather than to perform a quantitative meta-analysis or systematic review.

## TLSS AND RELATIONSHIPS TO DRUG RESISTANCE IN CRC

### Composition of TLS and implications for drug resistance

Structurally, TLSs are organized similarly to SLOs, featuring a central B cell zone (predominantly CD20+ B cells) surrounded by a T cell zone containing CD4+ helper T cells, CD8+ cytotoxic T cells, and DCs^[10,22]^. Unlike SLOs, TLS lack a surrounding capsule, enabling rapid migration of T cells and high-affinity antibodies into tumor tissues to exert anti-tumor effects. Within CRC, specific B cell subsets, including IGHD+ naïve B cells and CD27+ memory B cells, are predominantly localized within TLS^[23]^.

The maturation status of TLS, classified by two- or three-grade systems, critically influences their immunological function. Histologically, TLS range from simple T- and B-cell aggregates to organized structures with primary follicles or secondary follicles containing GCs^[24]^. The presence of GCs - comprising dark zones of Ki67+ proliferating B cells and light zones where B cells interact with CD21+CD23+ FDCs and T follicular helper (Tfh) cells - drives enhanced B cell maturation, memory B cell formation, plasma cell differentiation, and production of high-affinity antibodies^[25-29]^. Consequently, mature TLS with GCs correlate with better clinical prognosis and increased response to immune ICIs and CTLA-4 blockade^[30]^. The T cell zone contains CD4+ and CD8+ T cells, along with smaller populations of macrophages, DCs, and regulatory T cells (Tregs)^[31-34]^.

HEVs, characterized by MECA-79 and PNAd expression, are present within TLS in most primary CRC tumors. HEVs facilitate lymphocyte trafficking by supporting the entry of naïve T cells (CCR7+, CD44-) and central memory T cells (CD45RA-, CD62L+) into TLS^[35-37]^. Tumors with high HEV density exhibit increased infiltration of both CD3+ T cells and CD20+ B cells, promoting adaptive immune responses.

**Figure 1:**
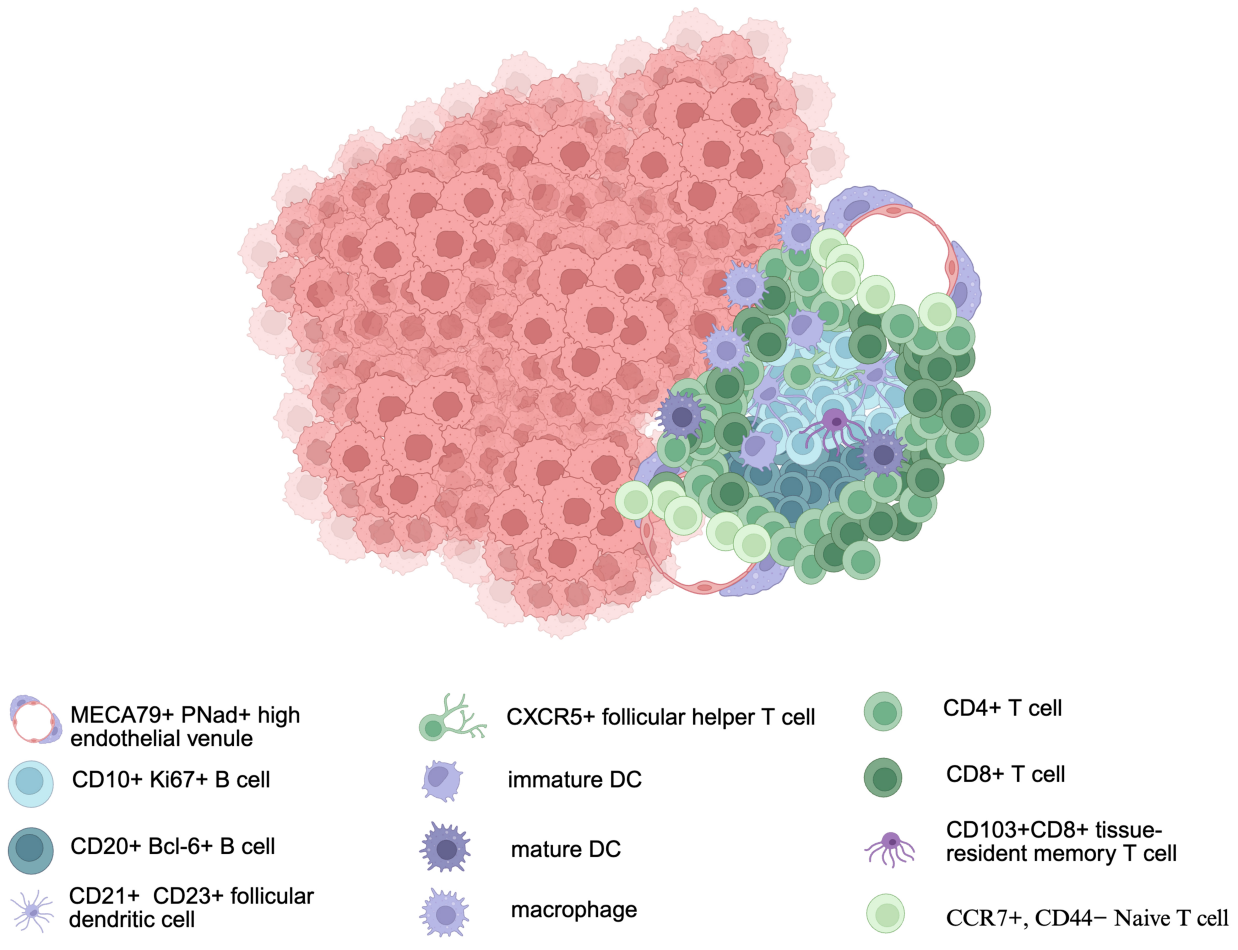
Detailed cellular composition and mature construction of TLS in CRC. This schematic delineates the architectural arrangement and key immune cell subsets within a mature TLS in CRC. Copyright permission link: https://BioRender.com/h060tsk. TLS: Tertiary lymphoid structure; CRC: colorectal cancer; MECA79: MECA-79 antigen; PNad: peripheral node addressin; CD: cluster of differentiation; CXCR: CXC chemokine receptor; DC: dendritic cell; CCR: CC chemokine receptor.

From a drug-resistance perspective, the structural and cellular composition of TLS may influence therapeutic outcomes. Mature TLS with fully formed GCs and functional HEVs support sustained anti-tumor immunity by generating high-affinity antibodies and facilitating effective T cell recruitment - mechanisms that are often compromised in drug-resistant tumors. Conversely, TLS that lack GCs or exhibit an enriched Treg compartment may contribute to an immunosuppressive microenvironment that promotes immune evasion and resistance to immunotherapy. The absence or immaturity of HEVs can impair lymphocyte trafficking, limiting the infiltration of effector T cells into the tumor and reducing sensitivity to ICIs. Thus, the compositional features of TLS - particularly the presence of GCs, functional HEVs, and balanced immune cell subsets - serve not only as prognostic indicators but also as contributors of therapeutic susceptibility, highlighting TLS as potential targets for overcoming drug resistance in CRC. Notably, the cellular and structural integrity of these structures is closely associated with therapeutic sensitivity. Tumors lacking these organized components often display immune exclusion, T-cell exhaustion, and impaired TLS maturation, which are clinically correlated with poor responses to conventional chemotherapy and ICIs. Detailed cellular composition and mature construction of TLS in CRC are presented in Figure 1.

### Maturation classification and implications for drug resistance of TLS in CRC

Based on a synthesis of previously published literature, the maturation classification of TLS can be broadly categorized into two-grade or three-grade systems, which reflect different understanding in organizational architecture, cellular composition, and genetic heterogeneity. The two-grade classification, primarily reported in esophageal squamous cell carcinoma (ESCC) and oral squamous cell carcinoma (OSCC), distinguishes between Agg-TLS (lymphocytic aggregates without FDCs or lymphoid follicles) and Fl-TLS (presence of at least one lymphoid follicle)^[38,39]^. In contrast, the three-grade classification is more commonly applied in CRC. In non-metastatic CRC (nmCRC, stage II-III), the three maturation stages are defined by progressive changes in cellular composition, particularly increasing frequencies of FDCs and mature B cells^[40]^. In CRC liver metastases (CRCLMs), TLS maturation is characterized primarily by organizational structure^[41]^. By integrating these two classification frameworks with histological and immunofluorescence features, a unified three-tier classification emerges: (1) early TLS (Agg), consisting of dense lymphocytic aggregates without differentiated FDCs or vascularization, negative for CD21 and CD23 expression; (2) primary follicle-like TLS (PFL-TLS), featuring closely packed clusters of predominantly naïve B cells surrounding a central FDC network (CD21+) but lacking GCs (CD23-); and (3) c TLS (SFL-TLS), characterized by the presence of GCs with HEVs, a T-cell zone containing mature DCs, and a GC comprising FDCs and B cells (CD21+CD23+). Notably, the gene expression profile associated with mature TLS includes not only chemokines with established roles in TLS formation and function (e.g., CXCL13, CXCL11, CXCL10) but also genes such as TGFB2 and VEGFB, which are linked to an immunosuppressive TME and may paradoxically inhibit TLS development. Collectively, the presence of GC is considered a prerequisite for mature TLS^[42-44]^. Detailed classification criteria are summarized in Tables 1 and 2.

**Table 1:** CRC-specific TLS maturation stages^[40-44]^

**TLS maturation stage**	**Histological definition**	**Key immunohistochemical markers**
Early TLS^[40,42]^	Dense lymphocytic aggregates, without FDCs or vascularization	CD21-, CD23-
PFL-TLS^[41-44]^	Central FDC network, surrounded by naïve B cells	CD21+, CD23-
SFL-TLS^[41-44]^	Presence of GCs (FDCs and B cells), with HEVs, T-cell zone	CD21+, CD23+, chemokines (CXCL13, CXCL11, CXCL10, *etc.*)

CRC: Colorectal cancer; TLS: tertiary lymphoid structure; FDCs: follicular dendritic cells; CD: cluster of differentiation; PFL-TLS: primary follicle-like tertiary lymphoid structure; SFL-TLS: secondary follicle-like tertiary lymphoid structure; GCs: germinal centers; HEVs: high endothelial venules.

**Table 2:** Classification and prognostic value of TLS maturation^[40,41,43,45-48]^

**TME**	**Classification standard**	**Prognostic factor**	**Prognostic value**
nmCRC, stage II-III^[40]^	[1] Early TLS (E-TLS, dense lymphocytic aggregates without differentiated FDCs)	TLS maturation	Recurrence; sensitivity to ICI
	[2] PFL-TLS (B cell clusters with FDC network but without GCs)		
	[3] SFL-TLS (with GCs)		
CRCLM^[41,43]^	[1] Aggregates (Agg): vague clusters of lymphocytes	Intratumor region of TLS	Clinical outcome
	[2] Lymphoid follicles I (Fol-I): lymphoid follicles without GCs formation		
	[3] Lymphoid follicles II (Fol-II): lymphoid follicles with GCs formation		
Six common gastrointestinal cancers^[45,48]^	[1] Lymphoid aggregates	TLS with GC	Independent prognostic factor
	[2] Primary follicles		
	[3] Secondary follicles with a GC		
ESCC, OSCC^[46,47]^	[1] Agg-TLS: tumors with only Agg and no Fl	Not directly transferable to CRC without validation	
	[2] Fl-TLS: tumors with at least one Fl structure		

TLS: Tertiary lymphoid structure; TME: tumor microenvironment; nmCRC: non-metastatic colorectal cancer; FDCs: follicular dendritic cells; PFL-TLS: primary follicle-like tertiary lymphoid structure; GCs: germinal centers; SFL-TLS: secondary follicle-like tertiary lymphoid structure; ICI: immune checkpoint inhibitor; CRCLM: colorectal cancer liver metastase; ESCC: esophageal squamous cell carcinoma; OSCC: oral squamous cell carcinoma.

Furthermore, the organizational architecture of TLS and its corresponding immunological functionality can be categorized into distinct maturational classes with differential implications for drug resistance. Immature TLS, characterized by loose aggregates of T and B cells with sparse DCs, fail to support efficient anti-tumor immune responses and are instead associated with a T-cell-exhausted, inflamed, and immunosuppressive TME - features that collectively contribute to therapeutic resistance. In contrast, mature TLS (mTLS) exhibit well-organized structures in which mature DCs engage in active interactions with T cells, and immune hubs - such as Tfh cells expressing CD4, PD-1, and CXCR5 - establish productive contact with B cells, positioning mTLS as central anti-tumor immune niches within the TME. Accumulating evidence indicates that mTLS serve as important factors in the tumor immune contexture and carry significant prognostic and potential predictive value for treatment response. Notably, in non-metastatic stage II and III CRC, the presence of immature TLS (including early TLS and PFL-TLS) exhibits a strong association with increased recurrence risk, whereas patients with low densities of mature TLS (SFL-TLS) face a significantly higher cumulative risk of recurrence^[40,49-51]^. These observations underscore the functional divergence between immature and mature TLS in shaping immune competence and suggest that promoting TLS maturation may represent a viable strategy to overcome immune resistance and improve clinical outcomes in CRC.

### Density, location, and heterogeneity of TLS as prognostic and drug resistance-related factors in CRC

In nmCRC, TLS are predominantly located in the interstitial or peritumoral regions and are composed mainly of B and T cells. TLS density is notably higher in right-sided colon cancer (RCC) and serves as an independent prognostic factor for 5-year OS, comparable to vascular invasion and AJCC stage^[52,53]^. Spatially, TLS can be classified as intratumoral (In-TLS), peritumoral (P-TLS), or distant-tumoral (D-TLS), with ongoing debate regarding their functional roles. In nmCRC, low P-TLS density - particularly when associated with immature TLS - correlates with reduced recurrence-free survival (RFS) and OS, independent of TNM stage, whereas In-TLS shows no significant prognostic value, possibly due to a higher proportion of Tregs within In-TLS^[54,55]^. This spatial heterogeneity extends to other cancer types and metastatic settings, underscoring the need for context-specific evaluation.

The prognostic and resistance-related implications of TLS are further complicated by their structural heterogeneity. Highly vascularized TLS containing HEVs and organized TLS with GCs are associated with increased infiltration of CD3+ T cells and enhanced anti-tumor immunity, predicting better outcomes in stage II CRC and other solid tumors^[56-59]^. Conversely, TLS with immature features or immunosuppressive cellular compositions may contribute to therapeutic resistance.

In CRCLMs, the relationship between TLS location and prognosis reverses compared to nmCRC: In-TLS correlates with superior outcomes, while P-TLS is associated with poor survival. Multiplex immunohistochemistry reveals that intratumoral TLS harbor higher densities of Tregs, M2 macrophages, and Tfh cells, all of which significantly influence RFS and OS^[60]^. Notably, recent evidence indicates that both mature In-TLS and P-TLS enriched with IgG+ plasma cells are linked to improved clinical outcomes, whereas TLS- areas are characterized by IgA+ plasma cells. The chemokines CCL19 and CXCL13 are enriched in TLS+ regions, with CCL19-producing cancer-associated fibroblasts (CAFs) promoting B cell recruitment via the CCL19-CCR7 axis, thereby facilitating TLS formation and the generation of therapeutic antibodies that may help overcome drug resistance^[61]^. Additionally, TLS presence in CRCLM is positively associated with specific KRAS mutation subtypes, suggesting a molecular link between TLS formation and resistance mechanisms^[62]^.

In CRC pulmonary metastases (CRCPM), TLS correlate with increased immune cell infiltration, though their prognostic value remains controversial. Notably, the density and distribution of tumor-infiltrating lymphocytes (TILs) and TLS are consistent between pulmonary metastases and primary tumors, with CD3+, CD8+, CD45RO+, and FoxP3+ TILs evident in both sites^[63-65]^. This consistency suggests that TLS-associated immune landscapes may be preserved during metastasis and could represent stable determinants of treatment response.

Overall, the density, location, and structural maturity of TLS collectively shape their prognostic significance and influence drug resistance. Understanding these heterogeneous features is essential for leveraging TLS as both a predictive biomarker and a therapeutic target to overcome resistance in CRC.

### Nomograms integrating TLS for predicting clinical outcomes and drug resistance level in CRC

Accumulating evidence supports that TLS density, location, and maturation status serve as independent prognostic factors in CRC, alongside traditional parameters such as TNM stage and tumor budding. Based on multivariate Cox regression analyses, several nomograms incorporating TLS features have been developed to enhance prognostic accuracy and, importantly, to reflect underlying mechanisms of therapeutic resistance.

One nomogram integrating TLS density, AJCC stage, and venous invasion accurately predicts 3- and 5-year OS in RCC, demonstrating superior predictive performance compared with models that use AJCC stage or TLS density alone^[53]^. Another nomogram incorporating P-TLS density, tumor stroma percentage, and TNM stage effectively predicts RFS and OS in nm CRC, with improved accuracy over TNM staging alone^[66]^. Notably, the inclusion of P-TLS density - which reflects immune competence at the tumor margin - may capture immune-mediated resistance mechanisms that are not accounted for by anatomical staging.

At the structural level, a nomogram incorporating the proportion of HEVs within TLS has been proposed. A high HEV/TLS ratio correlates with favorable prognosis, enhanced recruitment of CD3+ and CD8+ T cells and M1 macrophages, and reduced angiogenesis, suggesting that HEV-rich TLS facilitate effective anti-tumor immune infiltration and may overcome immune exclusion - a key mechanism of resistance to immunotherapy^[67]^. Conversely, low HEV expression alone does not confer prognostic benefit, underscoring the importance of TLS integrity in mediating immune function.

In the context of curative resection, TLS combined with low fibroblast activation protein (FAP) expression in the central tumor region is associated with significantly improved RFS, whereas high FAP expression in TLS-absent tumors predicts poor outcomes. This interaction suggests that TLS may counteract the immunosuppressive effects of CAFs, thereby mitigating resistance to adjuvant therapies^[68]^.

At the molecular level, a 12-chemokine signature (including CCL2, CCL3, CCL4, CCL5, CCL8, CCL18, CCL19, CCL21, CXCL9, CXCL10, CXCL11, and CXCL13) has been linked to TLS expression and immune infiltration. High expression of this signature is associated with RCC, high TLS density, BRAF mutation, CIMP-high status, MSI-H status, increased tumor-infiltrating cytotoxic T lymphocytes and myeloid DCs, and reduced recurrence rates^[69-72]^. These chemokines are directly involved in lymphocyte recruitment and TLS organization; their coordinated expression may therefore serve as a surrogate for a functionally competent TLS that can sustain anti-tumor immunity and prevent resistance. Conversely, loss or downregulation of this chemokine network may contribute to immune evasion and poor therapeutic response.

Collectively, these TLS-integrated nomograms - spanning clinical, structural, and molecular dimensions - offer refined prognostic stratification and provide insights into immune-mediated resistance mechanisms. By capturing the functional status of TLS, these models may help identify patients who are more likely to benefit from immunotherapy and other treatments, thereby guiding strategies to overcome drug resistance in CRC. Although TLS-integrated nomograms show promising stratification performance, the level of evidence varies. For example, the TLS density and venous invasion model achieved multi-center validation via propensity score matching, enhancing its reproducibility. Conversely, other models (e.g., HEV/TLS ratio) remain constrained by retrospective, single-center designs. Consequently, extensive cross-cohort validation in diverse populations and integration into prospective trials (e.g., EMPIRE or TAYLOR) are essential before clinical guideline adoption; the nomogram is shown in Table 3.

**Table 3:** Nomograms integrating TLS features for predicting clinical outcomes and patient recurrence in CRC^[52,53,67-69]^

**Detailed nomogram model**	**Prognostic indicator**	**Cancer type**	**Validation status/level of evidence**
Density of TLS, AJCC stage and venous invasion^[53]^	3-year OS, 5-year OS	RCC	Retrospective, multi-center validated (propensity score-matched)
P-TLS density, TSP and TNM stage^[52]^	2-year OS, 5-year OS	nmCRC	Retrospective, single-center; requires multi-center verification
HEV/TLS ratio, TLS^[67]^	OS and DFS	CRC	Retrospective, single-center cohort; requires prospective validation
FAP expression in central tumor, TLS^[68,69]^	RFS	CRC and OSCC	Retrospective cohort; lacks prospective clinical adoption trial

TLS: Tertiary lymphoid structure; CRC: colorectal cancer; AJCC: American Joint Committee Cancer; OS: overall survival; RCC: right-sided colon cancer; P-TLS: peritumoral tertiary lymphoid structure; TSP: tumor stroma percentage; TNM: Tumor, Node, Metastasis; nmCRC: non-metastatic colorectal cancer; HEV: high endothelial venule; DFS: disease-free survival; FAP: fibroblast activation protein; RFS: recurrence-free survival; OSCC: oral squamous cell carcinoma.

### Association of TLS with MSI-H or high-TMB CRC and implications for drug resistance

In CRC, the formation and maturation of TLS - historically described as Crohn’s-like lymphoid reactions - are significantly more prevalent in MSI-H tumors and those harboring BRAF, KRAS, CIMP-high, or HER2-amplified mutations compared with MSS or low-mutant tumors^[73]^. This enrichment is largely attributable to the high TMB characteristic of MSI-H CRC, which generates abundant neoepitopes that drive sustained immune activation. Consequently, the TME of MSI-H CRC exhibits heightened infiltration of activated natural killer (NK) cells, CD8+ T cells, and T helper cells^[74]^.

B cell-rich TLS, characterized by robust antigen presentation, accumulation of IgG+ plasma cells, and perforin-expressing B cells, are prominently observed in MSI-H and high-TMB CRC patients^[23,75]^. These TLS are further supported by CXCL13+ TH1-like cells, which recruit CCR5+ cells and promote TLS formation^[76]^. Additionally, TIM4 and FOLR2 double-positive macrophages localized within the T-cell zones of MSI-H-associated TLS correlate with high infiltration of B cells and effector CD8+ T cells, underscoring their role in sustaining productive anti-tumor immunity^[77]^. High-TMB tumors also exhibit elevated expression of lymphotactin 1 and 2 (XCL1 and XCL2), which facilitate cDC1 recruitment and T cell proliferation^[78]^.

Recent single-cell analyses have identified a distinct gene signature in MSI-H TLS, including high expression of class I MHC genes, chemokines that attract memory T cells (CXCL9, CXCL10), T cell-activating cytokines (IFNG), and factors supporting T and NK cell proliferation (IL15, CXCL16). These molecules serve as important drivers of TLS formation and immune activation^[79-87]^.

From the perspective of drug resistance, the robust TLS formation observed in MSI-H CRC represents a key factor of enhanced sensitivity to ICIs. In contrast, MSS CRC - characterized by lower TLS prevalence, reduced immune infiltration, and an immunosuppressive TME - exhibits intrinsic resistance to immunotherapy. Notably, the molecular features that drive TLS formation in MSI-H tumors, including high chemokine expression and antigen presentation capacity, are frequently absent or suppressed in MSS tumors, contributing to immune evasion and poor treatment response. Thus, TLS not only serve as a distinguishing immunological hallmark between MSI-H and MSS CRC but also represent a potential contributor of ICI sensitivity. Strategies aimed at inducing TLS formation in MSS tumors may therefore offer a promising approach to overcome intrinsic drug resistance and expand the therapeutic benefits of immunotherapy to a broader CRC patient population. Here, the gene signatures of MSI-H TL in CRC are summarized in Table 4, and specific cells in CRC are described in Table 5, respectively.

**Table 4:** Specific gene signatures in TLS of MSI-H CRC^[79-86]^

**Researchers**	**Gene names**	**Functional roles of upregulated genes**
Becht *et al.*^[79]^	*CXCL9*, *CXCL10*, *CXCL16*, *IFNG*, *IL15*	Production of IFN-γ, activation of NK cells
Kim *et al.*^[80]^	*CTSC*, *CCL5*, *CCL18*, *CXCL9*	Recruitment of immune cells, naïve T cells
Wang *et al.*^[81]^	*GBP2*, *CXCL10*, *CXCL11*	Simulators of IFN, infiltration of TILs
Masuda *et al.*^[82]^	*CXCL13*, *CD4*, *HAVCR2*	Formation of TLS, negative regulator of anti-tumor immunity
Mlecnik *et al.*^[83]^	*IL15*, *IFNG*, *GNLY*, *CCL3*, *CXCL16*	Production of IFN-γ, enrichment of NK cells and CD8+ effector T cell
Xu *et al.*^[84]^	*AEN*, *CD3E*, *TRGC2*, *ULBP3*	Enrollment of ILCs, response to ICI
Yang *et al.*^[85]^	*CCL19*, *CCL21*, *CXCL13*, *CCR7*	Formation of TLS, activation and recruitment of Lti cells
Jia *et al.*^[86]^	*KRAS*, *B2M*, *CTSC*, *IKZF3*, *CCL5*	Recruitment of immune cells, differentiation, proliferation of B cells

TLS: Tertiary lymphoid structure; MSI-H: microsatellite instability-high; CRC: colorectal cancer; NK: natural killer; TILs: tumor-infiltrating lymphocytes; CD: cluster of differentiation; ILCs: innate lymphoid cells; ICI: immune checkpoint inhibitor; Lti: lymphoid tissue inducer.

**Table 5:** Representative immune cell marker signatures associated with CRC TLS^[26,32,44,45,87-91]^

**Cell in CRC TLS**	**Main cell markers**	**Representative markers / functional annotation**
**B cells^[26,32,89-91]^**		
CD20+ naïve B cells	IGHD, FCER2, TCL1A, IL4R	
CD27+ memory B cells	CD27, AIM2, TNFRSF13B	
CD38+ GC B cells	BANK1, TNFRSF13C, MKI67, AICDA	Proliferation and somatic hypermutation (MKI67, AICDA)
CD138+ plasma B cells	MZB1, DERL3, TNFRSF17, JCHAIN	
**T cells^[44,45,87,88]^**		
Naïve T cells (Tn)	CCR7, LEF1, SELL, CD27, CD28	
Central memory T cells (T-CM)	CCR7, SELL, IL7R, PTGER2	Memory maintenance and lymphocyte trafficking
Effector memory T cells (T-EM)	GZMK, CXCR4, CXCR3, CD44	
Effector T cells	GNLY, NKG7, TBX21, PRF1, CX3CR1	
Recently activated effector memory or effector T cells (T-EMRA)	KLRG1, CX3CR1, FCGR3A, NKG7, PRF1, GNLY	S1PR1, S1PR5 and ITGB7: capability to circulate in the periphery and home to normal mucosa and tumors
CD4+ T cells		
Th1 cells	IFNG, TIGIT, ZEB2, ICOS	
- GZMK+ Th1 cells	GZMK, IFNG, CXCR3	
- CXCL13+ Th1 cells	CXCL13, BHLHE40, PDCD1, HAVCR2	
Th2 cells	IL13, CD69, GZMK, NKG7	
TH17 cells	IL23R, RORC, IL17A, FURIN	
Follicular T helper cells	CXCR5, BCL6, ICA1, TOX, TOX2	
Follicular T regulatory cells	FOXP3, IL10, IL2RA, CXCR5, PDCD1, IL10	
Treg cells	FOXP3, IL2RA, IKZF2	
- Tumor-Treg	FOXP3, CCR8, TNFRSF18, LAYN	
- Blood-Treg	FOXP3, IL2RA, CXCR5, PDCD1, IL10	
CD8+ T cells		
Mucosal-associated invariant T (MAIT) cells	SLC4A10, KLRB1, ZBTB16, NCR3, RORC, RORA	
Cytotoxic T cells	GZMK, PRF1, HOPX, LAG3, GZMA, GZMB	
Naïve CD8+ T cells	TCF7, SELL, ITGAE, IL2RA	
Intraepithelial lymphocytes		
Tissue-resident memory T cells (TRM)	CD69, ITGAE, CXCR6	
- Lamina propria TRM	CD6, CXCL1, MYADM, CAPG	
- Intraepithelial lymphocytes (IELs)	CD160, KIR2DL4, TMIGD2, KLRC1/2/3	Highly expressed NK cell markers
CTLA4+ Treg	IL32, KLF6, HLA-DRB1, ETS1, IL7R, KMT2E	
CTLA4- Tregs	TOX2, ANKRD17	
Exhausted CD8+ T cells (T-EX)	HAVCR2, CXCL13, PDCD1, LAYN, TOX	tumor exclusivity
Myeloid cells^[88-91]^		
Monocyte	C1QA, C5AR1, CCL19, CCL23	
- CD16-monocytes	CD16, FCN1, and VCAN	
- CD14-monocytes	CD14, IFITM2, LST1	
TAM macrophage		
- SPP1-macrophages	SPP1, VEGFA, FCN1	
- FOLR2-macrophages	FOLR2, MARCO, C1QA	
- CXCL5-macrophage	MMP1, CXCL2, S100A8	
DCs	XCR1, CLEC9A, CD11c	Antigen processing- and presentation-related pathway
NK cells	NKG7, PTPRC, CST7, DNAM-1	

CRC: Colorectal cancer; TLS: tertiary lymphoid structure; CD: cluster of differentiation; GC: germinal center; NK: natural killer; Treg: regulatory T cell; TAM: tumor-associated macrophage; DCs: dendritic cells.

### Conclusion: TLSs as biomarks in drug resistance of CRC

#### Predictive biomarkers

TLS serves as an indispensable predictive biomarker for therapeutic responsiveness, particularly in partitioning responses to ICIs. In MSI-H or high-TMB CRC, sustained chemokine networks (e.g., CXCL13, CCL19) drive robust TLS neogenesis and GC formation, rendering these tumors highly sensitive to immunotherapy. In stark contrast, MSS tumors typically exhibit low TLS prevalence and defective maturation, representing a hallmark of intrinsic immunotherapy resistance^[40,53,56]^.

#### Functional mediators of resistance

In addition, TLS functions as active functional mediators of resistance in CRC. Structurally mature TLSs equipped with functional HEVs and active GCs serve as productive local immune hubs. They facilitate high-affinity antibody production and the entry of tumor-reactive stem-like CD8+ T cells, effectively counteracting immune exclusion and mitigating drug resistance. However, when tumors actively disrupt these structures - resulting in HEV deficiency, a lack of GCs, or an enrichment of immunosuppressive cells like Tregs and LAMP3+ DCs. Notably, LAMP3+ DCs exhibit a context-dependent dual role throughout the TLS life cycle. Structurally, mature LAMP3+ DCs secrete homing chemokines (CCL19/CCL21) to guide naïve T cells and support early lymphoid aggregation. However, within the heavily suppressed microenvironment of conventionally resistant MSS rectal cancers, these cells shift toward a regulatory phenotype. In this therapy-refractory context, they upregulate immune checkpoint ligands and secrete anti-inflammatory cytokines (IL-10 and TGF-β). These factors may interfere with the CXCL13-mediated B-cell homing pathway and impair local T-cell activation, acting as potential negative regulators that may contribute to maintaining TLS in an immature state. Consequently, these observations should be interpreted with caution, and future studies are required to clarify the precise molecular mechanisms underlying these context-dependent effects^[40,67,88]^.

## FORMATION AND MATURATION OF TLS: MECHANISMS AND IMPLICATIONS FOR DRUG RESISTANCE

Despite ongoing investigations into the precise mechanisms underlying TLS generation and development, the fundamental prerequisites for TLS formation in tumors are increasingly well-defined. TLS arise in settings of chronic inflammation, sustained by persistent expression of lymphoid chemokines - particularly CXCL13, CCL19, and CCL21 - and are closely associated with high TMB. Tumor cells contribute by presenting antigens derived from aberrant gene expression, mutated oncogenes (e.g., KRAS, BRAF), MSI-H status, epigenetically modified self-molecules, cancer-testis antigens, and virally encoded proteins^[92-94]^. Given their structural and functional similarity to SLOs, TLS are thought to share core developmental mechanisms with SLOs, with both tumor cells and the tumor stroma - particularly CAFs - playing critical roles in inducing and localizing TLS formation^[92-94]^.

From the perspective of drug resistance, the presence and maturity of TLS serve as key contributors to therapeutic sensitivity. Tumors with high TMB and sustained chemokine expression - such as MSI-H CRC - exhibit robust TLS formation, which in turn supports durable anti-tumor immunity and favorable responses to ICIs. Conversely, tumors lacking these TLS-promoting features, including the majority of MSS CRC, often exhibit impaired TLS development, immune exclusion, and intrinsic resistance to immunotherapy. Moreover, the tumor stroma can exert dual effects: while certain stromal components facilitate TLS organization, others may suppress TLS formation through immunosuppressive signals. Understanding the mechanisms relevant to TLS formation - from chemokine signaling to stromal interactions - is therefore essential for developing strategies to induce TLS neogenesis in resistant tumors and expand the clinical benefits of immunotherapy to a broader patient population.

### Chemokine signaling pathways in TLS formation and their implications for drug resistance

#### CXCL13-CXCR5 axis

CXCL13 is an important chemokine driving TLS formation and localization. Induced by TGF-β and IL-7/21, CXCL13 is expressed by various cell types, including CD4+ TILs (Tfh, Tph, Th1), tumor-reactive CD8+ T cells, FDCs, HEVs, type 3 innate lymphoid cells (ILC3s), and even CAFs and tumor cells^[53,95-97]^. In CRC, CXCL13-expressing CD4+ and CD8+ T cells are frequently observed near carcinoma cells, particularly in dMMR tumors^[98,99]^. CXCL13 acts through its receptor, CXCR5, to recruit B cells and lymphoid tissue inducer (LTi) precursors, initiating TLS formation^[23,100-104]^. Neutralization of CXCL13 or its loss results in reduced infiltration of B cells and Tfh cells, impairing TLS development^[23,100]^.

#### Lymphotoxin-mediated positive feedback

CXCL13 recruits CXCR5+ LTi cells expressing lymphotoxin-α1β2 (LT-α1β2), which interact with LTβR on mesenchymal lymphoid-tissue organizer (LTo) cells. This interaction activates canonical and non-canonical NF-κB signaling, promoting LTo cells to upregulate chemokines (CXCL13, CCL19, CCL21) and adhesion molecules (ICAM-1, VCAM-1, MAdCAM-1), thereby facilitating further lymphocyte recruitment via HEVs^[105-112]^. Subsequently, LTi cells express TRANCE, activating TRANCER signaling that induces LT-α1β2 and IL-7 expression, establishing a positive feedback loop that expands lymphatic vessels, enhances FDC networks, and amplifies chemokine expression, ultimately supporting TLS organization and maturation^[113,114]^.

#### CCL19/21-CCR7 axis

CCL19 and CCL21, secreted by LAMP3+ DCs, HEVs, cancer cells, and fibroblastic reticular cells (FRCs), recruit naïve and central memory T cells expressing CCR7 and CD62L^[115-117]^. These T cells are guided to T-cell zones, while CXCR5+ B cells are recruited to B-cell zones in response to CXCL13, enabling GC formation and T cell–B cell cooperation essential for productive anti-tumor immunity^[118-121]^.

#### CCL28-CCR10 axis

The CCL28-CCR10 axis, primarily involving epithelial cell-derived CCL28, mediates the migration of IgG+ and IgA+ CCR10+ plasma cells from TLS peripheries into the tumor stroma, supporting humoral anti-tumor responses^[23,122]^. Additionally, CCR10+ CD4+ T cells enriched in IL-17- and IL-22-producing subsets (Th22 cells) suggest a role in T cell homing within the CRC TME^[123,124]^.

#### Implications for drug resistance

These chemokine networks are central to TLS formation and function and are directly relevant to drug resistance. Tumors with high expression of TLS-associated chemokines (e.g., CXCL13, CCL19, CCL21) exhibit enhanced lymphocyte infiltration, organized TLS, and improved sensitivity to ICIs. Conversely, loss or downregulation of these chemokine axes - often observed in MSS CRC or following immune evasion - impairs TLS formation, leading to diminished T and B cell recruitment, reduced GC activity, and compromised anti-tumor immunity. This chemokine-deficient state contributes to an immune-excluded or immune-desert phenotype that underlies resistance to immunotherapy and other treatments. Therefore, strategies aimed at restoring or enhancing the chemokine signaling pathways may represent a promising approach to promote TLS neogenesis and overcome drug resistance in CRC.

### Functional roles of tumor necrosis factor superfamily members in TLS formation and drug resistance

The tumor necrosis factor (TNF) superfamily, including TNF-α, TNF-β, CD40L, and LIGHT, plays key roles in TLS formation through complex interactions involving LTα1β2/LTβR signaling.

#### LTα1β2/LTβR axis

LTαβ expression on activated CD4+ T cells is essential for DC function, while LTβR signaling in DCs provides maturation signals downstream of CD40 activation^[125-127]^. For CD8+ T cells, the LT pathway supports clonal expansion but not effector function, whereas the CD40 pathway is required for CD8+ T cell effector function and induces CD86 expression on DCs^[125-127]^. LTα1β2/LTβR interactions between B cells and follicular DCs induce CXCL13 and CCL19 production, promoting DC and CD4+ T cell recruitment that supports helper T cell and Tfh responses^[128-130]^.

#### TNF-α, LIGHT, and cellular contributors

TNF-α secreted by tumor-associated neutrophils (TANs) recruits B cells to the TME and promotes their differentiation into plasma cells, a process mediated in part by membrane-bound BAFF on TANs^[131,132]^. LIGHT (TNFSF14), produced by Tfh cells, induces CCL21 production by tumor endothelial cells and fibroblasts; upon binding LTβR, LIGHT promotes blood vessel normalization and triggers intratumoral TLS formation^[74,133,134]^. Emerging evidence suggests that CD8+ T cells, NK cells, and M1 macrophages may function as alternative LTi cells in TLS via LTα3-TNFR signaling or TNF-α expression^[135-137]^.

#### TGF-β in T cell recruitment and retention

TGF-β1, abundant in epithelial tissues, plays a crucial role in CD8+ T cell recruitment and retention. TGF-β binding to its receptor promotes integrin-linked kinase (ILK) phosphorylation and AKT activation, inducing CD103 expression on CD8+ T cells. This facilitates T cell homing to the TME and enhances CCR5 recruitment at the immunological synapse between TILs and tumor cells^[138-141]^. TGF-β signaling also induces CD8α expression in intestinal T cell populations via the Smad2/Smad3 pathway, contributing to local immune surveillance^[142,143]^.

#### Implications for drug resistance

The TNF superfamily members and TGF-β signaling pathways that are associated with TLS formation have direct implications for drug resistance. Functional LTα1β2/LTβR and CD40/CD40L signaling are essential for productive DC-T cell interactions and sustained anti-tumor immunity; their disruption can lead to impaired TLS maturation and reduced ICI sensitivity. TNF-α and LIGHT promote B cell recruitment and vascular normalization, respectively, both of which enhance immune cell infiltration and therapeutic responsiveness. Conversely, TGF-β exhibits dual roles: while it supports CD8+ T cell retention via CD103 induction, excessive TGF-β signaling in the TME can promote immunosuppression, Treg differentiation, and resistance to immunotherapy. Thus, the balance of TNF superfamily and TGF-β signaling within TLS is strongly associated with the functional quality of anti-tumor immunity. Therapeutic strategies that modulate these pathways - such as LTβR agonists to enhance TLS maturation or TGF-β blockade to overcome immunosuppression - may represent promising approaches to modulate drug resistance and improve treatment outcomes in CRC.

### Interactions and crosstalk in TLS formation: HEVs, microbiota, and implications for drug resistance

#### HEVs as gatekeepers of TLS function

HEVs are specialized postcapillary venules that facilitate lymphocyte transmigration into TLS. In human and murine solid tumors, including CRC, HEVs express MECA-79 and peripheral node addressin (PNAd), enabling lymphocyte homing via L-selectin-, chemokine-, and integrin-mediated interactions^[138,144]^. HEVs express chemokines CCL19, CCL21, and CXCL13, which recruit T cells, DCs, and B cells, respectively, into organized TLS compartments^[145,146]^. Lymphocyte integrins - particularly αLβ2 (LFA-1) binding to ICAM-1/ICAM-2, and α4β7 binding to MAdCAM-1 - mediate firm adhesion and trans endothelial migration^[147-149]^. HEVs also serve as entry sites for CD62L+ T cells, including tumor-reactive PD-1+ Tcf1+ stem-like CD8+ T cells, which are important for sustained anti-tumor immunity and response to ICB^[150,151]^. Upregulated genes such as TSPAN7 and MEOX2 on tumor HEVs regulate lymphocyte extravasation and inhibit excessive angiogenesis, respectively^[152,153]^.

#### Microbiota-driven TLS formation

Gut microbiotas play an active role in inducing TLS in CRC. In orthotopic mouse models, *Helicobacter hepaticus* colonization promotes TLS formation with organized B- and T-cell zones, upregulates TLS-associated genes (e.g., *Lta*, *Tnfsf14*, *Icam1*), and enhances anti-tumor immunity^[154,155]^. *Bifidobacterium* augments DC function and CD8+ T cell priming, while *Akkermansia muciniphila* and *Enterococcus hirae* modulate IFN-γ and IL-2 expression, improving the efficacy of PD-1/PD-L1 inhibitors^[156-158]^. Additionally, CX3CR1hi macrophages enriched in TLS during *Salmonella* invasion secrete CXCL13, TGF-β1, and IL-10, contributing to TLS organization^[159-162]^.

#### Implications for drug resistance

HEVs and microbiota may contribute to TLS functionality with direct relevance to drug resistance. High HEV density and maturation correlate with enhanced recruitment of stem-like CD8+ T cells, which are essential for sustained responses to ICB and are often depleted in resistant tumors^[150,151]^. Conversely, HEV deficiency or immaturity impairs lymphocyte trafficking, contributing to immune exclusion and poor therapeutic response. The presence of specific gut microbiota that promote TLS formation - such as *H. hepaticus*, *Bifidobacterium*, and *A. muciniphila* - has been associated with improved ICI efficacy, whereas dysbiosis may undermine TLS integrity and foster resistance^[156-158]^. Thus, HEV integrity and favorable microbiota composition are emerging as key contributors of TLS-mediated anti-tumor immunity. Strategies aimed at enhancing HEV maturation (e.g., via LIGHT signaling) or modulating gut microbiota (e.g., probiotics or fecal microbiota transplantation) represent promising approaches to augment TLS function and overcome drug resistance in CRC.

**Figure 2:**
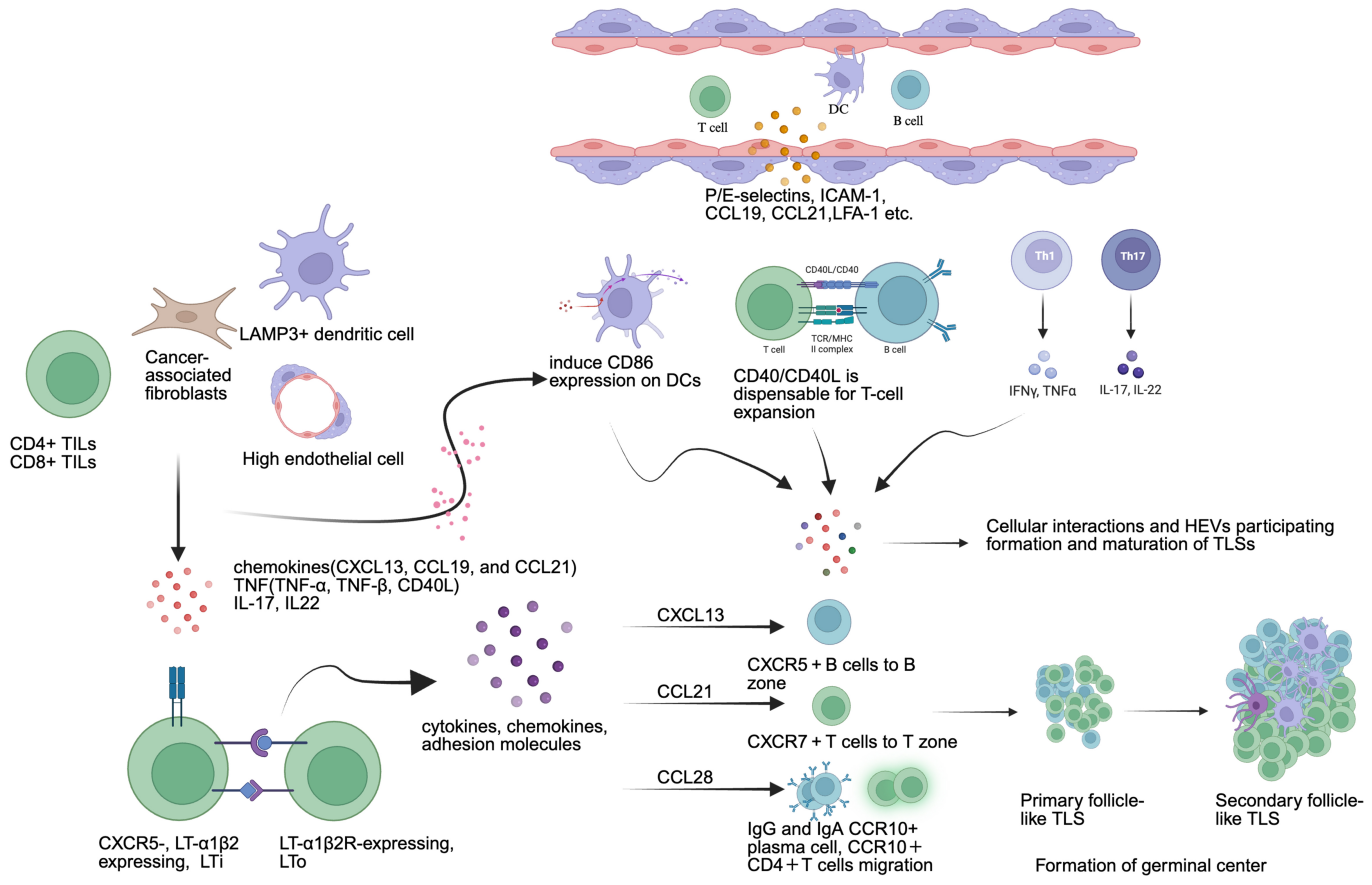
Mechanisms of formation and maturation of TLS in CRC. This diagram illustrates the cellular interactions, chemokine cascades (CXCL13, CCL19, CCL21), and TNF superfamily signaling pathways that drive the progressive development from early lymphocytic aggregates to mature SFL-TLS. Copyright permission link: https://BioRender.com/omsjn1g. TLS: Tertiary lymphoid structure; CRC: colorectal cancer; CXCL/CCL: CXC/CC chemokine ligand; TNF: tumor necrosis factor; SFL-TLS: secondary follicle-like tertiary lymphoid structure; DC: dendritic cell; ICAM-1: intercellular adhesion molecule 1; CD: cluster of differentiation; CD40L: CD40 ligand; IL: interleukin; CXCR: CXC chemokine receptor; LTi: lymphoid-tissue inducer cell; LTo: lymphoid-tissue organizer cell; HEVs: high endothelial venules.

Taken together, these chemokine networks coordinate the molecular pathways and cellular interactions that collectively influence the functional quality of anti-tumor immunity. Impaired chemokine axes or the accumulation of regulatory components (such as LAMP3+ DCs) frequently leads to immature TLS formation, thereby fostering an immune-excluded phenotype and driving therapeutic resistance. The mechanisms of formation and maturation of TLS in CRC are illustrated in Figure 2.

## CORRELATION OF TLS AND DRUG RESISTANCE IN CRC

### Molecular heterogeneity and TLS as modulators of oncogenic resistance

The genomic and transcriptomic heterogeneity of CRC imposes a profound barrier to therapeutic efficacy, frequently steering tumors toward chemoresistance and post-adjuvant recurrence. Genomic stratification suggests that the prognostic weight and functional configuration of TLS are intimately linked with specific oncogenic drivers. For instance, tumors harboring oncogenic BRAF mutations (BRAF^mut^) typically present an aggressive clinical phenotype refractory to standard chemotherapy. However, clinical evidence indicates that robust TLS formation can significantly attenuate this genetic disadvantage; BRAF^mut^ patients exhibiting high peritumoral or intratumoral TLS densities demonstrate OS rates comparable to their BRAF^mt^ counterparts. A parallel paradigm emerges in the context of targeted therapies for KRAS-driven and EGFR-mutant colorectal malignancies. While combined EGFR and KRAS^G12C^ inhibition represents a validated approach to counter adaptive resistance mechanisms in primary CRC, the structural integrity of the local immune microenvironment remains an important contributor to long-term durable responses. In CRCLMs, a high TLS score strongly correlates with specific KRAS mutation subtypes, characterized by an enrichment of IgG^+^ plasma cells and robust production of antitumor antibodies. Furthermore, quantitative nomograms confirm that TLS density and higher HEV proportions stand as independent favorable prognosticators for disease-free survival (DFS) in both EGFR-mutant and KRAS-mutant gastrointestinal tumors, irrespective of baseline PD-L1 tumor proportion scores. These insights highlight a novel therapeutic strategy: combining oncogenic kinase inhibitors with TLS-inducing interventions to synergistically dismantle cell-intrinsic resistance and reinforce cell-extrinsic immune clearance^[163-165]^.

#### BRAF mutations and TLS as a resistance modulator

BRAF mutations, prevalent in CRC, typically confer high-level drug resistance and poor clinical outcomes. However, compelling evidence shows that BRAF-mutant (BRAF^MT^) patients with high TLS expression and BRAF wild-type (BRAF^WT^) patients with low TLS expression share comparable OS, indicating that robust TLS formation may partially mitigate the adverse prognostic impact in BRAF-mutant CRC patients. Further analysis of TLS features - including location, number, and maturity - in relation to BRAF mutation status suggests that intratumoral TLS may enhance prognosis and sensitize BRAF^MT^ CRC patients to therapeutic interventions^[166]^.

#### EGFR signaling, KRAS mutations and TLS as a strategy to improve therapeutic sensitivity

Recent research identifies EGFR signaling as the dominant mechanism of CRC resistance to KRAS G12C inhibitors; correspondingly, dual targeting of EGFR and KRAS G12C effectively circumvents this resistance in CRC, reverting resistance to KRASG12C inhibition in CRC patients^[167,168]^. Similarly, in colorectal cancer liver metastases, the location and density of TLS are associated with molecular subtypes and clinical outcomes. The study found that the TLS score was positively correlated with specific KRAS mutation subtypes^[30]^. Meanwhile, TLS density has been confirmed as a potential prognostic biomarker for DFS in EGFR-mutant tumors, including CRC and lung adenocarcinoma, independent of PD-L1 TPS or EGFR mutation subtype, suggesting a potential therapeutic strategy to improve therapeutic sensitivity of patients with EGFR- or KRAS-mutation G12C CRC^[160,169,170]^.

#### Dynamic single-cell insights into TLS functionality

Single-cell mapping of immune landscape dynamics during CRC progression reveals significant functional differences in CD4+ Tfh and germinal center B (GC-B) cells between early and advanced CRC. Notably, CXCL13 expression on CD8+ Tex cells, along with CD40–CD40L interactions between CD4+ Tfh and B~GC~ cells, emerges as a key regulator of TLS functionality. These interactions significantly influence the response to immunotherapy and the development of drug resistance^[171]^.

#### Implications for overcoming resistance

Collectively, these findings underscore that TLS heterogeneity - shaped by molecular drivers such as BRAF mutations, immune cell interactions, and dynamic changes during disease progression - plays a pivotal role in determining therapeutic sensitivity. Strategies aimed at preserving TLS integrity, promoting B cell-mediated humoral immunity, or enhancing key interactions such as the CD40–CD40L axis may represent novel approaches to overcome drug resistance in CRC, particularly in molecularly defined subsets with poor baseline prognosis.

### Interactions between TLS and neoadjuvant drug treatment resistance

#### Functional roles of TLS in MSS CRC neoadjuvant drug treatment resistance

In MSS CRC, which typically exhibits limited response to chemotherapy or immunotherapy, recent studies reveal that responders harbor a higher abundance of more activated and mature TLS compared to non-responders. Importantly, LAMP3+ DCs have been identified as negative regulators of TLS formation in MSS CRC, suggesting that suppressing this cell subset may reduce drug resistance^[88]^. Similar findings in ovarian carcinoma and non-small cell lung cancer demonstrate that chemotherapy-induced endoplasmic reticulum stress drives TLS formation and maturation in metastatic lesions, thereby increasing infiltration of ICI-responsive TCF1+CD8+ T cells^[172]^. Neoadjuvant chemotherapy also upregulates B-cell- and TLS-associated genes (JCHAIN, CXCR4, MS4A1), correlating with enhanced antigen presentation and deeper pathological remission, including complete remission^[173]^. Neoadjuvant chemoradiotherapy further promotes TLS maturity within tumors and improves OS. Radiotherapy modulates the TME to activate and proliferate immune cells within mature TLS - including CD20+Ki-67+ B cells, CD21+ DCs, CD8+Ki-67+ cytotoxic T cells, and CD4+Ki-67+ Th cells - thereby enhancing immunotherapy efficacy^[174,175]^.

#### Functional roles of TLS in advanced CRC neoadjuvant drug treatment resistance

In advanced CRC, multivariate analysis confirms that the presence of TLS in primary tumors prior to systemic chemotherapy is significantly associated with favorable chemotherapy response and prolonged survival. Mature TLS correlates with MAdCAM-1+ vessels and higher CD3+/CD8+ T-cell infiltration in both primary and metastatic tumors^[176]^. Based on these insights, a multi-center TLS gene signature has been established. A high TLS score - characterized by increased proportions of CD8+ T cells, B cells, and DCs, along with activation of anti-tumoral signaling pathways - predicts better response to neoadjuvant FOLFOX chemotherapy and sintilimab (PD-1 blockade), even in MSS CRC^[177]^. Similarly, the presence of mature TLS with GCs, enriched for activated CD4+ memory cells, naïve B cells, and NK cells, forms in the context of high-quality tumor neoantigens, leading to enhanced humoral immunity that is sensitive to neoadjuvant therapy in gastrointestinal solid tumors^[178,179]^.

#### Implications for drug resistance

These findings collectively demonstrate that TLS maturation status is strongly associated with sensitivity to neoadjuvant therapies. TLS deficiency or immaturity represents an actionable mechanism of drug resistance. Key resistance-reversing strategies include: (1) suppressing or modulating LAMP3+ DCs to block their IL-10/TGF-β signaling axes, thereby removing the molecular brakes on chemokine cascades to restore mature TLS formation in MSS CRC; (2) Harnessing chemotherapy-induced ER stress to promote TLS neogenesis; (3) Combining radiotherapy to enhance TLS maturity and immune cell activation; (4) Utilizing TLS gene signatures to identify resistant patients who may benefit from alternative or combination regimens. These approaches hold promise for overcoming intrinsic resistance, particularly in MSS CRC populations that are conventionally refractory to treatment.

## MEASURES FOR INDUCING TLS TO OVERCOME DRUG RESISTANCE AND IMPROVE THERAPEUTIC EFFICACY OF CRC

As the presence of TLS is associated with improved ICI response and reduced cancer drug resistance, it is hypothesized that the enhancement of TILs represents a pivotal therapeutic strategy, which may be achieved via inducing the formation of TLS, by accelerating the maturation of TLS or by enriching the density of TLS. Increasing studies suggest that the presence of TLS within the TME is associated with favorable prognoses and a higher likelihood of responding to ICB therapy in several cancer types^[180]^.

### Inducing TLS to overcome drug resistance: enriching TILs and HEVs

Emerging evidence highlights that therapeutic strategies aimed at inducing TLS formation - particularly through enriching TILs and HEVs - hold promise for overcoming drug resistance in CRC.

#### Radiotherapy and immunotherapy combinations

In mouse solid tumor models, hypofractionated radiotherapy combined with lenalidomide (an immunomodulatory agent) induces TLS formation in a CD8+ T cell- and type I interferon-dependent manner. This combination enhances anti-tumor CD8+ T cell immunity, DC cross-presentation, upregulates TLS-associated genes (GZMB, IFN-γ, TNF-α), and increases tumor-associated HEV (TA-HEV) density. Importantly, it recruits and enriches stem-like (TCF1+ TIM3- PD1+) and transitory (TCF1- TIM3+ CD101- PD1+) tumor-specific T cell subsets, both of which are important for sustained responses to immunotherapy^[181,182]^. These findings suggest that radiotherapy-ICI combinations can overcome resistance by promoting TLS neogenesis and sustaining effector T cell pools.

#### Chemotherapy-induced TLS formation

Certain chemotherapeutic agents also demonstrate TLS-inducing potential. Combined administration of tanshinone IIA (TSA) and astragaloside IV (As) normalizes tumor blood vessels - including HEVs - while reducing immunosuppressive factors, thereby enhancing TIL abundance and activity^[183]^. This dual mechanism addresses two key resistance pathways: poor lymphocyte infiltration and an immunosuppressive microenvironment.

#### Nanovaccines and bio-nanoparticle strategies

Bio-nanoparticle-based λ phage vaccines targeting Aspartate β-hydroxylase (ASPH), a tumor-associated antigen, show promising TLS-inducing effects. When combined with PD-1 blockade, ASPH-targeting vaccines reduce immunosuppressive Treg infiltration and, upon PD-1/PD-L1 inhibition, promote CXCL13 production by ASPH+ cancer cells, which recruits CXCR5+ CD8+ T lymphocytes to TLS. This is accompanied by increased enrichment of effector and memory CTLs, Tfh cells, GC-B cells, and FDCs^[184]^. Similarly, lymph node-targeting nanovaccines enhance tumor immune responses and accelerate the tumor-immunity cycle^[185,186]^. These approaches directly counteract immune evasion mechanisms that underlie resistance to conventional immunotherapies.

#### 3D-printed scaffold vaccines

Implantable 3D-printed scaffold vaccines represent an innovative strategy for *in situ* TLS induction. These scaffolds recruit antigen-presenting cells (APCs) and T cells, initiating TLS formation with mature features, including activation of NK cells, macrophages, and CD11c+ DCs. When combined with PD-L1 blockade, scaffolds further upregulate the proliferation (Ki67+), cytotoxic (granzyme B+), and IFN-γ-producing CD8+ T cells^[187]^. Other scaffold designs incorporating ovalbumin, GM-CSF, and sustained TLR2 agonists facilitate long-term DC activation and recruitment of activated CD8+ cytotoxic T lymphocytes and memory T cells^[188,189]^.

#### Implications for drug resistance

Collectively, these TLS-inducing strategies address multiple mechanisms of drug resistance: (1) enhancing lymphocyte infiltration via HEV normalization and chemokine recruitment; (2) expanding stem-like and effector T cell populations that sustain anti-tumor immunity; (3) reducing immunosuppressive Tregs; and (4) generating durable memory responses. By converting immunologically “cold” tumors into “hot” tumors with organized TLS, these approaches hold potential to overcome intrinsic and acquired resistance to immunotherapy and other treatments in CRC and beyond.

### Stimulator of interferon genes agonists as crucial triggers for TLS induction and overcoming drug resistance in CRC

Stimulator of interferon genes (STING) agonism has emerged as a promising strategy to induce TLS formation and overcome drug resistance in CRC. Activation of the STING pathway within the TME upregulates both anti-angiogenic factors (e.g., Tnfsf15/VEGI, CXCL10) and TLS-inducing chemokines (e.g., CCL19, CCL21, LTA, LTB, LIGHT), leading to enhanced infiltration of CD8+ T cells and CD11c+ DCs, as well as local TLS neogenesis^[190,191]^.

#### Advanced STING-activating platforms

Innovative delivery systems have been developed to optimize STING-mediated TLS induction. A STING-activating hydrogel (ZCCG) incorporating Zn^2+^, chitosan (a STING pathway activator), and CpG (a TLR9 agonist) enables dual activation of cGAS-STING and TLR9 pathways. This dual-pathway approach effectively enhances immune cell infiltration and expression of lymphocyte-recruiting chemokines, thereby promoting TLS formation and potentiating tumoricidal immunity^[192]^. Mannose-modified stearic acid-grafted chitosan (M-CS-SA) micelles target the cGAS-STING pathway, increasing CD3+ CD8+ T cell infiltration and accelerating DC maturation^[193]^.

Additional STING-oriented strategies include cyclic dinucleotides combined with Pam3CSK4 (Pam3CSK4-CDGSF), a conjugated adjuvant targeting both STING and TLR1/2, as well as immunostimulatory nanoparticles (immuno-NPs) containing monophosphoryl lipid A (MPLA) and cyclic diguanylate (cdGMP)^[194,195]^. These platforms are designed to enhance TLS formation in tumors with high mutational burden and are summarized in Table 6^[190-195]^.

**Table 6:** STING agonists as therapeutic methods for TLS in TME^[190-195]^

**STING interventions**	**Mechanistic pathway**	**Induction roles in TLS and TME**
Low-dose STING agonist ADU S-100 (5 µg/mouse) (*in vivo*)^[190]^	TCRβ-CDR3 on TILs, LIGHT/TNFSF14, TLR7/9 on DCs	Enhanced infiltration of CD8+ T cells and CD11c+ DCs, local TLS neogenesis
ZCCG plus Zn^2+^ with 4,5-imidazole dicarboxylic acid (*in vivo*)^[192]^	cGAS/STING, CpG/TLR9	Enhanced infiltration of immune cells, expression of lymphocyte-recruiting chemokines, and induced formation of TLS
M-CS-SA micelles dose of 15 mg/kg plus oxaliplatin dose of 5 mg/kg (*in vivo*)^[193]^	cGAS/STING	Increased infiltration of CD3+ CD8+ T cell, maturation of DCs
Pam_3_CSK_4_-CDG^SF^ (*in vivo*)^[194]^	cGAS/STING, Pam_3_CSK_4_-CDG^SF^/TLR1/2	Increased production of Th1 cytokines (TNF-α, IL-6) and IFN-γ+CD8+ T cells, enhanced APC activation
Immunostimulatory nanoparticle (immuno-NP), (*in vivo*)^[195]^	cdGMP/STING, MLPA/TLR 4	Increased amount of innate immune cell subpopulations and APC, extended perivascular space

STING: Stimulator of interferon genes; TLS: tertiary lymphoid structure; TME: tumor microenvironment; TILs: tumor-infiltrating lymphocytes; DCs: dendritic cells; CD: cluster of differentiation; TNF: tumor necrosis factor; APC: antigen-presenting cell.

#### Implications for drug resistance

STING agonists address multiple mechanisms underlying therapeutic resistance. First, they promote vascular normalization via anti-angiogenic factors, counteracting the abnormal vasculature that limits immune cell infiltration in resistant tumors. Second, they upregulate chemokines that recruit T cells and DCs, overcoming immune exclusion - a hallmark of resistance to ICIs. Third, by inducing TLS formation, STING agonists establish sustained immune niches that support the generation of durable anti-tumor responses. Notably, tumors with low baseline TLS formation - such as MSS CRC - often exhibit poor responses to ICIs; STING activation represents a strategy to convert these immunologically “cold” tumors into “hot” tumors with organized TLS, thereby overcoming intrinsic resistance. Thus, STING-targeted therapies hold significant promise as a combinatorial approach to sensitize resistant CRC to immunotherapy and other treatments.

### Clinical applications and trials targeting TLS in CRC

Several clinical trials investigating TLS-associated strategies in CRC have been registered on ClinicalTrials.gov. One completed single-arm, open-label phase II trial evaluated the combination of regorafenib and avelumab in patients with advanced or metastatic solid tumors^[196]^. Published results demonstrated antitumor activity in biliary tract cancers (A phase II trial), with patients exhibiting high PD-L1 expression showing greater treatment benefit^[197]^. However, clinical data specifically assessing TLS in CRC patients remain limited, highlighting an urgent need for dedicated, systematic clinical trials that incorporate TLS as a biomarker or therapeutic target.

#### Implications for drug resistance

The current lack of TLS-focused clinical trials in CRC represents a significant gap, particularly given the established association between TLS and response to ICIs. In resistant tumor types - including MSS CRC, which comprises the majority of cases - the absence of mature TLS correlates with immune exclusion and poor ICI efficacy. Therefore, future trials should prioritize: (1) prospective evaluation of TLS as a predictive biomarker for immunotherapy response and resistance; (2) investigation of combination strategies (e.g., radiotherapy, STING agonists, or targeted therapies) designed to induce TLS formation in resistant tumors; and (3) inclusion of TLS-related endpoints to assess whether TLS neogenesis translates into improved clinical outcomes. Table 7 summarizes registered clinical trials involving TLS+ CRC cohorts^[196-200]^, underscoring the evolving but still nascent landscape of TLS-directed therapeutic strategies in CRC.

**Table 7:** Clinical trials based on TLS-associated strategy and treatment in CRC^[196-200]^

**Title**	**Conditions**	**Design and arms**	**Primary objective**	**Role of TLS**	**Phase**	**NCT number and results**
Targeting MDMD and PD1 in tumors with TLSs (EMPIRE)	Solid tumors (includes MSS CRC)	Single-arm: Ezabenlimab + BI907828	Disease control rate (DCR) within 24 weeks	Exploratory biomarker, roles in targeting PD1 and/or MDMD drug resistance	II	NCT06084689^[198]^ Results: not applicable (11/06/2025)
Phase Regorafenib + Avelumab	Advanced digestive solid tumors (includes CRC not MSI-H or dMMR)	Single-arm: Phase I: Regorafenib +Avelumab Phase II: Regorafenib (80 mg/day)	Confirmed objective response rate (ORR)	Positive antitumor activity in heavily pretreated biliary tract cancer population^[197]^	Ib/II	NCT03475953^[197]^ Results: not applicable (31/12/2025)
Combining epigenetic and immune therapy to beat cancer (CAIRE)	Metastatic solid tumors with TLS	Single-arm: Durvalumab + Tazemetostat	OS, PFS	Roles in drug resistance, enhanced immune therapy	II	NCT04705818^[199]^ Results: no CRC-specific results reported
MEDI5752 in patients with mature TLSs solid tumors (TAYLOR)	Patients with TLS+ solid tumors	Single-arm: patients with TLS+ PD1/PDL1-experienced solid tumor	Two-stage three-outcome	Anti-tumor efficacy and treatment response of the presence of mature TLS	II	NCT05888857^[200]^ Results: applicable (03/2026)

TLS: Tertiary lymphoid structure; CRC: colorectal cancer; MSS: microsatellite-stable; MSI-H: microsatellite instability-high; dMMR: mismatch repair deficiency; OS: overall survival; PFS: progression-free survival.

### Critical perspectives and future directions: risks of TLS targeting

Despite the therapeutic promise of inducing TLS to overcome drug resistance in CRC, potential risks and unanswered questions necessitate a balanced perspective.

#### Potential risks

First, TLS functionality is context-dependent. Immature or disorganized TLS may harbor regulatory T cells, M2 macrophages, or exhausted T cells, potentially fostering immunosuppression rather than anti-tumor immunity. Second, TLS-associated B cells can include IL-10-producing regulatory subsets that suppress effector T cell function. Third, therapeutic TLS induction - particularly with STING agonists or radiotherapy combined with ICIs - risks autoimmune toxicities such as colitis or pneumonitis. Fourth, TLS in metastatic sites has been linked to worse prognosis in some cancers, raising concern about unintended pro-metastatic effects.

#### Future research directions and toward a risk-benefit framework

Key open questions include: (1) How to selectively induce protective *vs.* immunosuppressive TLS? (2) What are the optimal timing and combination strategies for TLS neogenesis? (3) Can TLS be safely induced in patients with chronic inflammation or autoimmune predisposition? (4) Do TLS influence metastatic progression in CRC? (5) How can TLS assessment be standardized for clinical trials?

TLS-directed therapies should be considered context-dependent interventions. Future trials must incorporate safety endpoints addressing autoimmunity and metastasis, alongside efficacy measures. Patient stratification by baseline TLS status, molecular subtype (MSI-H *vs.* MSS), and immune landscape will be essential to maximize benefit and minimize risk.

#### Prospective cohort incident-tumor biobank method as a promising approach

Most current evidence on TLS in CRC derives from cross-sectional analyses of tumor specimens obtained at the time of diagnosis or surgery. While these studies have established important associations between TLS and clinical outcomes, they cannot address the temporal sequence of TLS development or distinguish whether TLS are a cause or consequence of effective anti-tumor immunity. Emerging research frameworks such as the prospective cohort incident-tumor biobank method (PCIBM) offer a promising approach to overcome these limitations^[201]^. By integrating long-term prospective follow-up of large populations with tumor tissue profiling of incident cancer cases, PCIBM enables the investigation of how decades of environmental exposures, lifestyle factors, and host characteristics influence the development and maturation of TLS within the TME^[202]^. For example, recent PCIBM-based studies have successfully linked long-term smoking habits with CRC incidence stratified by tumor mutational and neoantigen loads, providing unique etiological insights that would be impossible to obtain through traditional case-series designs^[203]^. We propose that future TLS research would greatly benefit from adopting similar prospective cohort approaches to examine the long-term factors of TLS formation and their causal role in modulating drug resistance.

## CONCLUSION AND LIMITATIONS IN TLS OF CRC

Positioned directly within the malignant stroma and anatomically mirroring SLOs, mature TLS function as key frontline sentinels in the antitumor immune landscape. It is now well established that the presence of structurally organized TLS - characterized by BCL6+ GC-B cells, CD21+CD23+ FDC networks, functional MECA-79+ HEVs, and a balanced infiltration of cytotoxic CD8+ T cells and CXCR5+ Tfh cells - serves as a powerful contributor of therapeutic sensitivity and prolonged survival in CRC. Mechanistic insights into the chemokine grids (CXCL13, CCL19, CCL21) and TNF superfamily cues that is associated with TLS ontogeny have illuminated translational pathways to potentially modulate drug resistance. Novel engineering modalities, including STING-activating hydrogel systems, bio-nanovaccines, 3D-printed scaffolds, and hypofractionated radiotherapy combinations, hold tremendous promise to therapeutically induce TLS neogenesis and sensitize traditionally refractory MSS CRC to immunotherapy.

Nevertheless, several important knowledge gaps must be bridged before TLS-targeted strategies can be fully integrated into clinical practice. A primary limitation of the current literature is the predominance of retrospective, cross-sectional studies, which capture merely a static snapshot of the TME and fail to establish whether TLS are a true causal driver or merely a bystander consequence of effective immunity. The dynamic evolution of these structures over the natural history of CRC remains poorly understood. To address this issue, future research must leverage robust prospective frameworks, such as the PCIBM, to trace how long-term host exposures and longitudinal treatments reshape TLS functionality. Additionally, the field urgently requires a standardized, consensus-based scoring system to uniformly categorize TLS maturation grades and cellular compositions across pathological laboratories. Sequentially and systematically conducted multicenter clinical trials are mandatory to clarify the risk-benefit ratios of therapeutic TLS induction, ensuring that induced structures do not inadvertently harbor immunosuppressive subsets like LAMP3+ DCs or FoxP3+ Tregs that drive autoimmunity or accelerate metastasis. Resolving these challenges will undoubtedly elevate TLS from valuable prognostic biomarkers to powerful tools in the personalized armamentarium against CRC resistance.

Although accumulating evidence supports an important association between TLS and therapeutic responsiveness in CRC, most available studies remain retrospective or preclinical. Future multicenter prospective studies and mechanistic investigations are required to validate TLS-based biomarkers and therapeutic strategies before routine clinical implementation.

## DECLARATIONS

### Copyright

© The Author(s) 2026.
